# Fuzzy Logic Approaches for Causal Inference in Health Care: Systematic Review

**DOI:** 10.2196/83425

**Published:** 2026-03-25

**Authors:** Jaime Jamett, Patricio Melendez, Ximena Collao-Ferrada, Karina Cordero-Torres, Alejandro Veloz

**Affiliations:** 1 Office of the Vice President for Academic Affairs, Universidad de Valparaíso, Blanco 951, Valparaíso, 2340000, Chile, 56 962069194; 2 PhD Program in Sciences and Engineering for Health, Universidad de Valparaíso, Valparaíso, Chile; 3 Department of Maxillofacial Radiology, Faculty of Dentistry, Universidad Andres Bello, Viña del Mar, Chile; 4 Department of Preclinical Sciences, School of Medicine, Universidad de Valparaíso, Valparaíso, Chile; 5 Interdisciplinary Center for Biomedical Research and Engineering for Health (MEDING), Universidad de Valparaíso, Valparaíso, Chile; 6 Interdisciplinary Center for Health Studies, Universidad de Valparaiso, Valparaiso, Chile; 7 School of Biomedical Engineering, Universidad de Valparaíso, Valparaíso, Chile

**Keywords:** fuzzy logic, causality, delivery of health care, clinical decision-making, health information systems

## Abstract

**Background:**

Fuzzy logic has been progressively investigated as a viable alternative to traditional statistical and machine learning methods in health care modeling, especially in environments marked by uncertainty, nonlinearity, and missing information. Although its use in prediction, classification, and risk stratification is well established, its application to explicit causal inference remains limited, varied, and methodologically premature.

**Objective:**

This systematic review aimed to examine how fuzzy logic frameworks have been used to address causal questions in health care, focusing on their methodological characteristics, comparative performance, and degree of integration with formal causal inference approaches.

**Methods:**

A systematic search across 6 databases (PubMed, Web of Science, ScienceDirect, SpringerLink, Scopus, and IEEE Xplore) identified peer-reviewed studies published between 2014 and 2025 that applied fuzzy modeling in health care settings with explicit or implicit causal objectives. The review adhered to PRISMA (Preferred Reporting Items for Systematic Reviews and Meta-Analyses) 2020 guidelines and used a modified PICO (population, intervention, comparator, and outcome) framework for study selection. Data were extracted on the health care domain, fuzzy method, comparator use, and causal framing. Risk of bias was evaluated using the Joanna Briggs Institute (JBI) checklist and the PROBAST+AI tool, according to study design.

**Results:**

A total of 37 studies met the inclusion criteria. The most frequently applied approaches were fuzzy inference systems, fuzzy cognitive maps, and neuro-fuzzy models, with applications spanning infectious diseases, cancer, cardiovascular health, mental health, and occupational health. Fourteen studies included comparator models; among these, 5 reported superior performance of fuzzy approaches, 3 showed comparable results, and 6 lacked sufficient detail for a robust comparison. Only 2 studies explicitly implemented formal causal inference frameworks, while most relied on predictive or associative modeling with implicit causal assumptions. Overall, the risk of bias was moderate to high.

**Conclusions:**

Fuzzy logic offers interpretability and flexibility well suited to complex health care problems, yet its application to explicit causal inference remains fragmented. Greater methodological transparency, systematic benchmarking, and integration with formal causal designs—such as counterfactual and target trial frameworks—are required to establish fuzzy logic as a robust paradigm for causal inference in health care.

## Introduction

Health care is strongly influenced by uncertainty. Clinical and public health decisions are routinely made under conditions of incomplete, ambiguous, or imprecise information, arising not only from individual patient variability but also from the complexity of health care systems and the processes through which real-world data are generated [1]. Such uncertainty encompasses both stochastic variability and epistemic constraints and is further amplified by heterogeneous, noisy, and nonlinear data from electronic health records, diagnostic imaging, physiological signals, and population-level monitoring systems [2]. Under these conditions, conventional statistical approaches—typically relying on fixed thresholds, linearity, and prespecified functional forms—often struggle to represent gradual transitions, ambiguous diagnostic boundaries, and context-dependent relationships that characterize real-world clinical data [1-3].

Causal inference emerged as a methodological approach for estimating the effects of exposures or interventions on health outcomes, explicitly addressing the limitations of purely associational analyses [4-10]. Rather than focusing on prediction, this approach centers on counterfactual questions—what would be expected to occur under hypothetical alterations in treatment or exposure—by making causal assumptions explicit and, in principle, empirically assessable [9,11-13]. This perspective is particularly relevant in health care and public health, where randomized controlled trials are frequently impractical and observational data constitute the primary source of evidence [9,11,13,14]. In such settings, causal reasoning is commonly formalized using directed acyclic graphs, which encode assumptions about causal structure, confounding, and intervention pathways, thereby enabling principled identification of causal effects [15-17].

Recent developments have emphasized the central role of explicit study design in strengthening causal inference from observational data. Target trial emulation (TTE) clarifies the causal question by prespecifying the key protocol components of a hypothetical randomized trial—including eligibility criteria, treatment strategies, time zero, follow-up, and outcomes—prior to analysis, thereby aligning observational studies with the core principles of randomized experiments [17-20]. While this design-oriented framework can reduce avoidable biases and enhance interpretability, it does not by itself ensure valid effect estimation. In practice, TTE still requires appropriate identification assumptions and estimation strategies and may remain vulnerable to challenges such as model misspecification or limited flexibility when representing complex data structures [17].

Despite advances in causal inference and design-oriented approaches, substantial challenges persist at the estimation stage when analyzing complex health care data. Even when causal questions are explicitly defined, commonly used estimation methods often rely on inflexible functional assumptions, sharply delineated variables, and correctly specified models—conditions that are difficult to sustain in high-dimensional and heterogeneous clinical environments [3,8,11]. As a result, a methodological gap remains between rigorously specified causal designs and the flexible representation of nonlinearity, gradual clinical thresholds, and uncertainty inherent in observational health data, limiting the applicability of traditional causal models in complex real-world settings [1,2,11,14,21].

As a response to the demand for flexible representations of uncertainty and nonlinearity, fuzzy logic has been adopted as a modeling paradigm in health care research, grounded in earlier theoretical developments on vagueness and graduality. Central to this evolution was Zadeh’s introduction of fuzzy sets as a generalization of classical set theory, in which membership is defined by degrees rather than binary inclusion [22-24]. This formulation provided a formal mathematical basis for representing ambiguity, partial truth, and gradual transitions in complex systems, thereby enabling the representation of phenomena that cannot be adequately captured using crisp categories. Building on this foundation, subsequent developments extended fuzzy sets into operational fuzzy logic systems, particularly through rule-based inference mechanisms that support reasoning with linguistic variables and imprecise conditions [25].

In clinical contexts, this representational flexibility facilitates the translation of gradual and linguistically defined clinical concepts into implementable computational models. Building on these foundations, a range of fuzzy logic–based approaches—including fuzzy inference systems (FIS), adaptive neuro-fuzzy inference systems (ANFIS), fuzzy cognitive maps (FCM), Takagi-Sugeno models, and fuzzy clustering—have been applied across diverse health care domains. These applications span infectious diseases [26-34], cardiology [35-42], oncology [43-49], obstetrics [50-52], mental health [53,54], and occupational health and safety [55-59].

More recently, fuzzy logic–based approaches have increasingly been combined with machine learning and artificial intelligence techniques to enhance predictive performance, scalability, and automation in health care applications [60,61]. Despite this growing convergence, the extent to which such hybrid models explicitly engage with causal reasoning—through the definition of counterfactual estimands, formal identification strategies, and transparent causal assumptions—remains inconsistently reported in the literature. Against this background, this systematic review aimed to evaluate and synthesize evidence on the application of fuzzy logic–based approaches for causal inference in health care.

## Methods

### Design and Reporting Standards

The review was conducted following established systematic review standards, with adaptations to accommodate computational health modeling studies [62]. Eligibility criteria and search strategy used a modified PICO framework, targeting complex datasets (Population), fuzzy logic for causal inference (Intervention), conventional modeling methods (Comparator), and performance or interpretability outcomes (Outcomes).

The review was conducted and reported in accordance with the PRISMA (Preferred Reporting Items for Systematic Reviews and Meta-Analyses) 2020 guidelines to ensure transparency and methodological rigor [63]. A systematic search was performed across 6 bibliographic databases, with references managed using Zotero (v7.0.15) and blinded title-abstract and full-text screening conducted in Rayyan (Qatar Computing Research Institute). The review protocol was prospectively registered in PROSPERO (registration number CRD420251044493). Risk of bias was assessed using the Joanna Briggs Institute (JBI) checklist for analytical cross-sectional studies [64] and the PROBAST+AI tool for predictive modeling studies [65]. The procedures applied at each stage of the review are described in detail below.

This systematic review addresses a critical gap in the literature regarding how fuzzy logic–based approaches have been used to support causal inference in health care. The primary research question guiding the review was: How have fuzzy modeling approaches been applied, alone or in combination with other methods, to address causal questions in complex, multivariable health care settings? Rather than testing superiority, the review aimed to examine the contexts, modeling strategies, and assumptions under which fuzzy logic–based methods have been used in relation to causal objectives, particularly in settings characterized by uncertainty, nonlinearity, and high-dimensional data.

### Research Questions and Scope

To structure this analysis, three secondary questions were defined: (RQ1) What modeling characteristics and design features are commonly reported in fuzzy-based approaches addressing causal questions? (RQ2) Under what data or problem contexts are fuzzy logic–based methods compared with conventional modeling approaches? (RQ3) How are the resulting insights framed in relation to clinical or policy-relevant decisions?

### Eligibility Criteria

Studies were eligible for inclusion if they applied fuzzy logic–based approaches in health care settings and demonstrated either an explicit or implicit causal objective. Explicit causal intent was defined using formal causal frameworks, counterfactual reasoning, or clearly articulated intervention contrasts. Implicit causal intent was identified when modeling structures, analytical interpretations, or conclusions were framed in terms of causal effects, intervention impact, or decision-relevant implications beyond prediction. This inclusive criterion allowed the review to capture both formally specified and informally articulated causal approaches.

Studies were excluded if they did not use fuzzy-based techniques, lacked any causal objective, or focused exclusively on diagnostic classification or prediction without causal interpretation.

### Information Sources and Search Strategy

The literature search was conducted between March and June 2025 across 6 electronic databases: PubMed, Web of Science, ScienceDirect, SpringerLink, Scopus, and IEEE Xplore. Search strategies combined controlled vocabulary terms (eg, MeSH [Medical Subject Headings] in PubMed) with platform-specific free-text keywords to capture studies at the intersection of fuzzy logic, causal inference, and health care. To enhance sensitivity, the search was intentionally broad and complemented by manual screening of reference lists from included studies. The full search strategies for each database are reported in Table 1.

**Table 1.** Search strategies across databases for the identification of relevant studies.

Database	Search strategy
PubMed	(“Fuzzy Logic” [MeSH] OR “Fuzzy logic” [Title/Abstract] OR “Fuzzy modelling” [Title/Abstract] OR “Fuzzy inference system*” [Title/Abstract]) AND (“Causality” [MeSH] OR “Causal Inference” [Title/Abstract] OR “Causal Model*” [Title/Abstract] OR “Causal Discovery” [Title/Abstract]) AND (“Healthcare” [Title/Abstract] OR “Medical Informatics” [MeSH] OR “Clinical Decision-Making” [MeSH] OR health* [Title/Abstract] OR clinical [Title/Abstract] OR medical [Title/Abstract])
Web of Science, ScienceDirect, Springer, IEE Xplore, and Scopus	(“fuzzy logic” OR “fuzzy modelling” OR “fuzzy inference system*”) AND (“causal inference” OR “causal model*” OR “causal discovery”) AND (health* OR clinical OR medical OR “medical informatics” OR “clinical decision*”)

### Study Selection Process

Eligible studies were required to be peer-reviewed, published in English between 2014 and 2025, and to provide sufficient methodological detail to allow critical appraisal. Only original research articles with accessible full text and direct relevance to clinical or health policy decision-making were included. While the reporting of performance metrics (eg, accuracy and area under the curve, AUC) and the use of comparator methods were encouraged, their absence did not constitute grounds for exclusion when studies provided substantive contributions to fuzzy modeling or causal reasoning in health care.

Studies were excluded if they were not written in English, did not constitute original research (including narrative or systematic reviews, editorials, commentaries, or conference abstracts without full text), were published outside the predefined time frame, involved extremely small samples (fewer than 5 observations), or lacked sufficient methodological transparency to support reproducibility or critical appraisal. These criteria were applied to ensure inclusion of studies with empirical relevance, conceptual rigor, and clarity in reporting.

Study selection was managed using Zotero and Rayyan (Qatar Computing Research Institute). Two reviewers (JJ and KC-T) independently screened titles, abstracts, and full texts according to predefined inclusion and exclusion criteria. Discrepancies were resolved through discussion with a third reviewer (PM). Interreviewer agreement was 94%, and final inclusion decisions were reached by consensus, with oversight provided by additional authors (XC-F and AV).

### Data Extraction and Classification

After removal of duplicates (n=6) and clearly irrelevant records (n=390), 225 records were retained for title and abstract screening. Of these, 153 were excluded based on predefined inclusion criteria, leaving 72 full-text articles assessed for eligibility. No reports were lost during retrieval. Thirty-five full-text articles were excluded, most commonly due to publication outside the predefined time frame (n=27), as well as wrong population (n=2), wrong outcome (n=3), wrong publication type (n=1), or wrong study design (n=2). A total of 37 studies were included in the final synthesis.

Following the inclusion of 37 studies, a structured data extraction process was implemented to ensure consistency while accommodating methodological heterogeneity. Two reviewers independently extracted data using a piloted extraction form, with discrepancies resolved through consensus or, when necessary, third-party adjudication. The extraction framework was designed to capture both technical modeling features and elements relevant to causal framing and interpretation.

### Evidence Synthesis

Extracted variables were organized across four domains: (1) bibliographic and contextual information (author, year, journal, and health care domain); (2) data characteristics (data source, dataset type, and sample size); (3) modeling and analytical features, including fuzzy modeling framework (eg, FIS, FCM, neuro-fuzzy, and Takagi-Sugeno), comparator methods (eg, generalized linear models, structural equation models, and directed acyclic graph–informed analyses), and reported performance metrics (eg, accuracy, AUC, and root mean square error); and (4) elements related to causal framing, including stated causal assumptions, interpretability features, and reported clinical or policy implications.

To support consistency across studies and reduce terminological heterogeneity, ELICIT—an artificial intelligence–assisted evidence synthesis platform—was used to standardize extracted terminology, assist in the classification of modeling approaches, and check internal coherence of extracted items. ELICIT was used as a supportive tool for data organization and synthesis and did not replace reviewer judgment in data extraction or interpretation.

### Risk of Bias Assessment

Risk of bias was assessed using two complementary tools, selected according to the methodological design of each included study. The JBI checklist [64] was applied to studies with observational or associational designs, particularly those using structural causal reasoning without formal identification strategies. Studies focused on predictive model development or validation were evaluated using the PROBAST+AI tool [65].

Tool-specific criteria guided the assessment of potential bias. For studies evaluated with PROBAST+AI, emphasis was placed on outcome definition, predictor handling, and analytical transparency. For studies assessed using the JBI checklist, particular attention was given to reporting adequacy, conceptual rigor, and overall methodological clarity. These assessments identified recurrent limitations related to internal validity and reporting quality across the included evidence.

Overall risk of bias was classified as low, moderate, or high based on the severity and frequency of methodological concerns identified using each assessment tool. Given the substantial methodological heterogeneity of the included studies, a formal GRADE (Grading of Recommendations Assessment, Development, and Evaluation) assessment was not performed. Instead, the certainty of the evidence was appraised qualitatively by triangulating risk of bias assessments, methodological coherence, and robustness of reporting.

### Data Synthesis and Analytical Strategy

Following data extraction, findings were synthesized to characterize modeling approaches, identify comparative trends, and highlight evidence gaps at the intersection of fuzzy logic and causal inference. Descriptive statistics were used to summarize the distribution of included studies by health care domain, modeling approach, data source, and sample size, with frequencies and proportions calculated for fuzzy modeling techniques, data types (real-world or synthetic), and reported performance metrics.

In parallel, a thematic analysis was conducted across 4 focal areas: diversity of modeling frameworks, comparative performance, treatment of causal assumptions, and relevance to clinical or policy decision-making. This analytical phase also identified recurring methodological limitations, reporting inconsistencies, and underexplored applications, thereby complementing the structured risk of bias assessments. Summary tables were used to support structured comparison across studies and ensure consistent classification by health care domain, fuzzy modeling technique, comparator method, and reported performance metrics.

## Results

**Figure 1.**
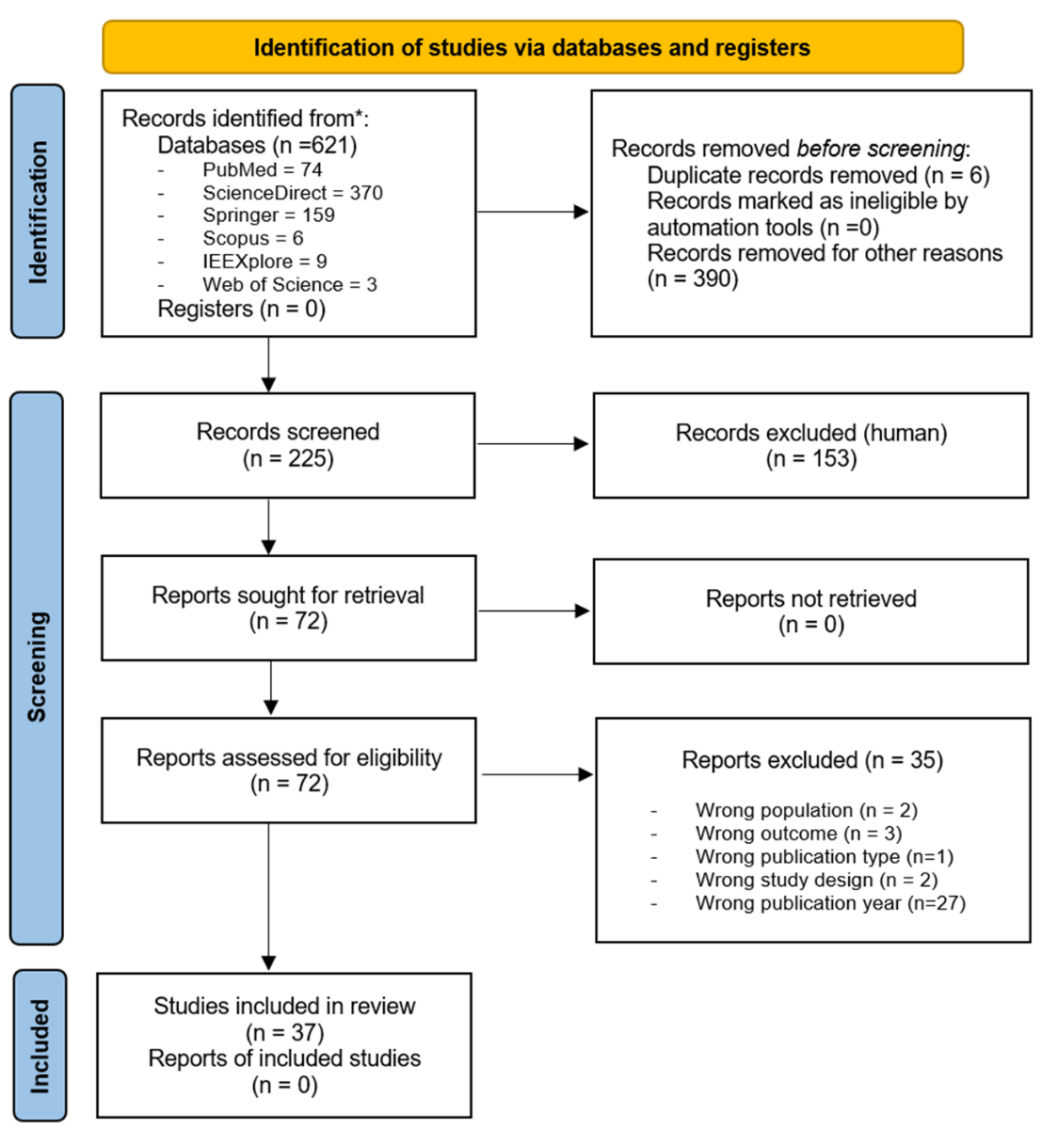
Flow diagram illustrating the identification, screening, eligibility assessment, and inclusion of studies in the systematic review, according to PRISMA (Preferred Reporting Items for Systematic reviews and Meta-Analyses) 2020 guidelines [63].

During the systematic search conducted between March and June 2025, a total of 621 records were identified across 6 electronic databases. After removal of duplicates and screening of titles, abstracts, and full texts, 37 studies published between 2014 and 2025 met the inclusion criteria and were retained for final synthesis. The PRISMA 2020 flow diagram (Figure 1) details the study selection process, including the number of records screened, excluded, and included at each stage of the review.

Table 2 lists fuzzy logic–based methodologies from 37 studies, showing the literature’s methodological diversity. The table defines each approach, its main analytical role, common application in reviewed studies, and frequency of use.

Table 3 summarizes the 37 studies included in the final synthesis, spanning health care domains such as infectious diseases, cardiovascular conditions, cancer, mental health, occupational health, and preterm birth. Across studies, the most frequently applied fuzzy approaches were FIS (Mamdani type), ANFIS, fuzzy analytic hierarchy process (FAHP) variants, and FCM. Sample sizes varied widely, ranging from fewer than 100 participants to large-scale public datasets exceeding 1000 cases, with data sources including institutional or hospital records, expert-based judgments, and simulated data.

Fourteen studies used direct comparator methods, most commonly logistic regression, decision trees, or ensemble classifiers, whereas the remaining studies relied on baseline comparisons or did not include external benchmarks. Performance was typically reported using accuracy or AUC, with sensitivity and specificity included in selected cases. Importantly, only a minority of studies explicitly framed their analyses within formal causal inference paradigms, while most remained primarily predictive or associative in scope.

**Figure 2.**
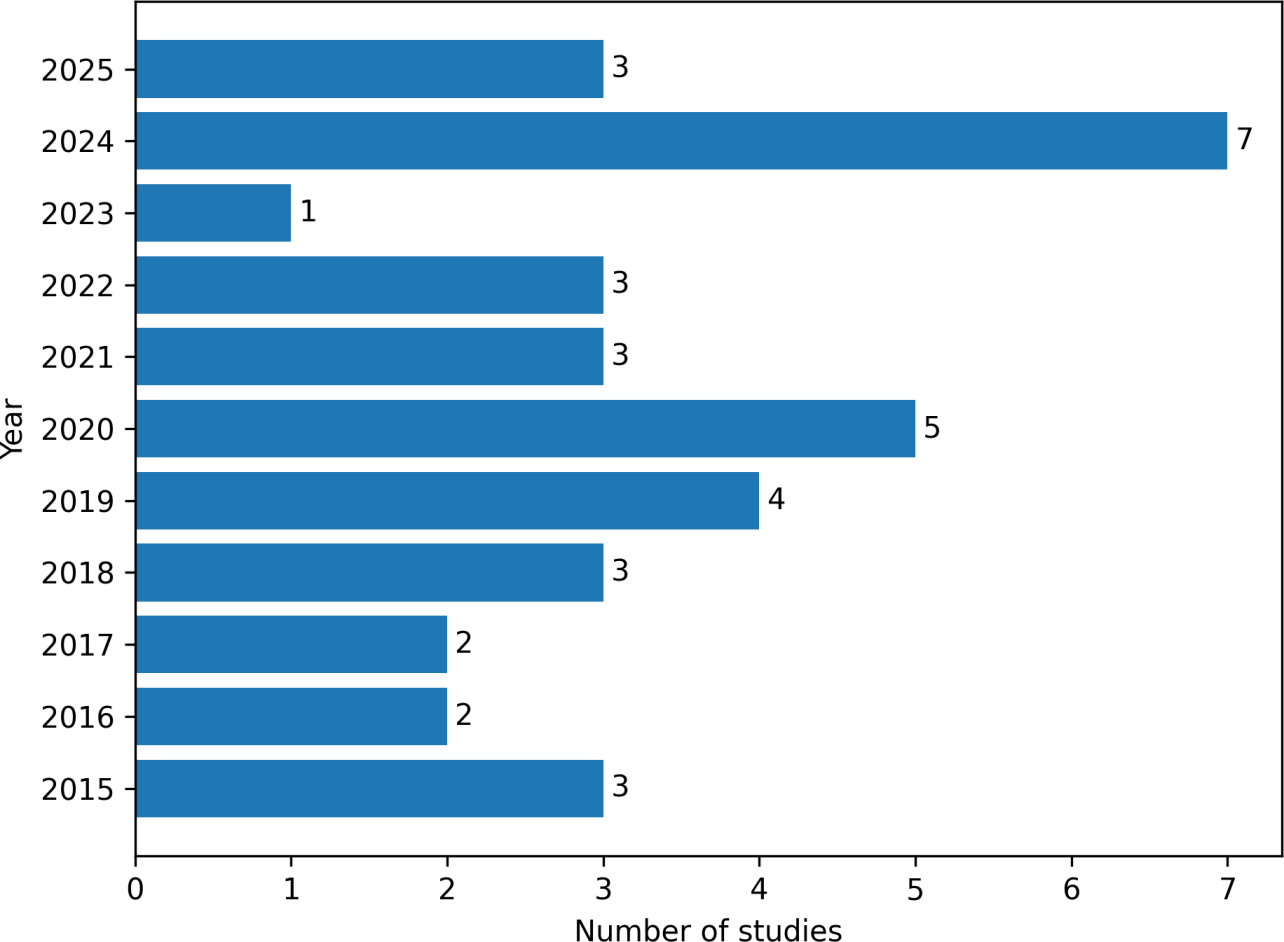
Chronological distribution of included investigations (2015‐2025). The bar chart illustrates the annual number of studies published throughout the review period.

The temporal distribution of the included studies shows an uneven pattern over the past decade, with episodic increases rather than a steady growth trajectory, culminating in a pronounced peak in 2024 (Figure 2).

**Table 2.** Fuzzy logic–based methods identified across the included studies (n=37)^a^.

Fuzzy method	Abbreviation	Primary analytical role	Typical application in reviewed studies	Studies, n (%)
Fuzzy inference system (Mamdani-type and variants)	FIS	Rule-based modeling under uncertainty	Prediction, classification, decision support	8 (21.62)
Fuzzy analytic hierarchy process	FAHP	Multicriteria decision analysis	Risk prioritization, decision support	6 (16.22)
Fuzzy cognitive maps	FCM	Conceptual modeling of interacting variables	Simulation of influence structures, exploratory causal reasoning	5 (13.51)
Adaptive neuro-fuzzy inference system	ANFIS	Hybrid learning and fuzzy inference	Prediction and pattern recognition	3 (8.11)
Hybrid fuzzy models combined with MCDM^b^	Hybrid Fuzzy + MCDM	Multicriteria decision support	Structural prioritization and ranking	3 (8.11)
Fuzzy clustering (C-means/K-means)	—^c^	Unsupervised pattern discovery	Grouping and exploratory analysis	2 (5.41)
Fuzzy-trace theory	FTT	Cognitive decision modeling	Behavioral and decision-making analysis	2 (5.41)
Fuzzy-set qualitative comparative analysis	fsQCA	Configurational causal analysis	Identification of necessary and sufficient conditions	2 (5.41)
Takagi-Sugeno fuzzy models	TS/TSK	Rule-based functional approximation	Predictive modeling	1 (2.70)
Fuzzy failure mode and effects analysis	F-MEA	Risk and failure assessment	Safety and risk analysis	1 (2.70)
Mediative fuzzy logic	MFL	Decision mediation modeling	Clinical decision support	1 (2.70)
Fuzzy evidential reasoning	FER	Evidence aggregation	Decision support under uncertainty	1 (2.70)
Likelihood-fuzzy analysis	LFA	Probabilistic-fuzzy integration	Risk estimation	1 (2.70)
Profile-based fuzzy association rule mining	PB-FARM	Pattern and rule discovery	Association analysis	1 (2.70)

a Some categories in this table group closely related fuzzy logic–based methods that are reported under different specific names across individual studies (Tables3  4). In particular, hybrid fuzzy models combined with multicriteria decision-making encompass approaches that integrate fuzzy rule–based systems with decision-analytic or influence-structuring techniques, such as decision-making trial and evaluation laboratory, analytic network process, analytic hierarchy process variants, or type-2 fuzzy sets. Similarly, several variants of fuzzy cognitive maps, neuro-fuzzy systems, and Takagi-Sugeno models are reported using study-specific nomenclature and are therefore grouped according to their underlying modeling principles. These groupings were applied to facilitate synthesis and comparability across studies with conceptually similar analytical objectives, while detailed methodological implementations, comparators, and performance metrics for each study are reported in Tables3  4.

b MCDM: multicriteria decision-making.

c Not applicable.

**Table 3.** Summary of characteristics and comparative outcomes of included studies.

Study (year)	Domain^a^	Task/outcome^b^	Fuzzy method^c^	Dataset size^d^	Data^e^	Comparator^f^	Primary metric^g^	CI^h^
Amirkhani et al (2014) [66]	Other	Autoimmune hepatitis	NFCM+NFIS	M	Inst	Direct: ANFIS	AUC 89.8	I
Lee et al (2015) [30]	ID	HIV prevalence (policy)	fsQCA	L	Pub	None	Consistency 0.95	E
Maranate et al (2015) [67]	Other	OSA severity	FAHP	L	Inst	None	Sens 92.3	P
Subramanian et al (2015) [43]	Cancer	Breast cancer risk	L2-FCM	S	Synth	Direct: FCM	AUC 94.3	P
Wolfe et al (2015) [49]	Cancer	Risk decision-making	FTT	M	Pub	Direct: RCT control	NR	P
Mollalo and Khodabandehloo (2016) [31]	ID	Leishmaniasis risk map	FAHP+GIS	L	Inst	Base	AUC 90.5	P
Yılmaz et al (2016) [45]	Cancer	Lung cancer	ANFIS-MEP	L	Inst	Direct: ANFIS, EP	AUC 94.6	P
Pota et al (2017) [47]	Cancer	Radiotherapy side effects	LFA	S	Inst	Direct: NB	AUC 0.81	P
Stanković and Stanković (2017) [48]	Cancer	Prostate survival	Neuro-fuzzy	S	Inst	Direct: ANN, FIS	*R*^2^=0.83	P
Iancu (2018) [42]	CVD	CVD diagnosis	MFL	NA	Synth	None	NR	P
Sabahi (2018) [39]	CVD	CHD risk ranking	BFAHP	NA	Exp	Direct: AHP	AUC 0.86	P
Saleh et al (2018) [41]	CVD	Diabetic retinopathy	ANFIS	M	Inst	Direct: RF, MLP, kNN	AUC 0.84	P
Argyropoulos et al (2019) [68]	Other	AKI stage-3 risk	TSK	L	Inst	Direct: LR	AUC 0.95	P
Romero et al (2019) [33]	ID	Dengue risk	FIS-Mamdani	NA	Pub	None	AUC >0.86	P
Sarkar et al (2019) [26]	ID	Malaria ecological risk	FIS+AHP	L	Mixed	Base	NR	P
Souza et al (2019) [51]	PTB	PTB phenotypes	Fuzzy clustering	L	Inst	None	NR	P
Boni et al (2020) [35]	CVD	CVD in dialysis	FIS-Mamdani	M	Inst	None	AUC 0.92	P
Hynek et al (2020) [54]	Mental	Refugee mental health	FCM	S	Exp	None	NR	I
Mahmoodi et al (2020) [44]	Cancer	Gastric cancer	FCM-NHL	M	Inst	Direct: ANN, SVM, DT, NB	AUC 95.8	I
Piyatilake and Perera (2020) [32]	ID	Dengue clusters	FAHP	L	Pub	Base	AUC 0.73	P
Malakoutikhah et al (2021) [58]	OHS	MSD risk (steel)	FIS-Mamdani	M	Mixed	None	*r*=0.24	P
Shi et al (2021) [34]	ID	Outbreak risk	FER	S	Exp	None	α=0.79	P
Yavari et al (2021) [40]	CVD	Heart disease profiling	PB-FARM	M	Pub	Direct	Conf 0.73	P
Mohandes et al (2022) [57]	OHS	Construction safety	IVIF-DEMATEL+ANP	S	Pub	Direct	α=0.74	E
Safaei et al (2022) [38]	CVD	Obesity model	MFRBS+DEMATEL	L	Pub	None	NR	I
Barbounaki and Sarantaki (2022) [50]	PTB	PTB risk assessment	FAHP	M	Inst	Base	NR	P
Brust-Renck and Reyna (2023) [46]	Cancer	Cancer risk decisions	FTT	L	Inst	Base	NR	P
Aydın and Özkan (2024) [36]	CVD	LMIC cardiovascular risk profiling	IVPF-AHP+TOPSIS	L	Inst	None	NR	P
Benito et al (2024) [28]	ID	COVID/dengue	FCM+LAMDA	L	Pub	Direct: RF, LAMDA	AUC 0.89	I
Chen et al (2024) [53]	Mental	Child depression	fsQCA+OLS	M	Mixed	None	Consistency 0.867	I
Costa et al (2024) [29]	ID	Leishmaniasis risk	FIS-Mamdani	L	Pub	None	NR	P
Sakinala et al (2024) [56]	OHS	Mining MSD risk	FIS-Mamdani	S	Inst	Base	*P*=.19	P
Sümbül-Şekerci et al (2024) [69]	Other	T2DM cognition	FCM+CRT	M	Inst	Direct: CRT	AUC 0.91	P
Upadhyay et al (2024) [55]	OHS	Iron ore MSD risk	FIS-Mamdani	S	Inst	None	NR	P
Demir and Sabır (2025) [59]	OHS	Workplace risk	F-FMEA	S	Exp	Direct: FMEA	NR	P
Rani and Dhanasekar (2025) [27]	ID	Zika risk factors	Type-2 FS+MCDM	NA	Exp	None	*r*>0.92	P
Scrobota et al (2025) [70]	ID	Periodontitis (T2DM)	FIS-Mamdani	S	Inst	None	*P*=.02	P

a ID denotes infectious diseases; CVD, cardiovascular diseases; OHS, occupational health and safety; and PTB, preterm birth.

b Task/outcome: OSA: obstructive sleep apnea; CVD: cardiovascular diseases; CHD: coronary heart disease; AKI: acute kidney injury; PTB: preterm birth; MSD: musculoskeletal disorders; LMIC: low- and middle-income countries; T2DM: type 2 diabetes.

c Fuzzy modeling approaches include fuzzy inference systems (FIS), adaptive neuro-fuzzy inference systems (ANFIS), fuzzy cognitive maps (FCM), fuzzy analytic hierarchy process (FAHP), fuzzy-set qualitative comparative analysis (fsQCA), Takagi-Sugeno-Kang models (TSK), fuzzy evidential reasoning (FER), fuzzy-trace theory (FTT), mediative fuzzy logic (MFL), and related hybrid extensions. neuro-fuzzy cognitive map (NFCM); geographic information system (GIS); t-norm modified Einstein operator (MEP); likelihood-fuzzy analysis (LFA); mediative fuzzy logic (MFL); bimodal fuzzy analytic hierarchy process (BFAHP); nonlinear Hebbian learning (NHL); profile-based fuzzy association rule mining algorithm (PB-FARM); interval-valued intuitionistic fuzzy decision-making trial and evaluation laboratory (IVIF-DEMATEL); Mamdani fuzzy rule-based system integrated with the decision-making trial and evaluation laboratory method (MFRBS-DEMATEL); interval-valued Pythagorean fuzzy analytic hierarchy process combined with the technique for order preference by similarity to ideal solution (IVPF-AHP-TOPSIS); fuzzy cognitive maps combined with learning algorithm for multivariate data analysis (FCM+LAMDA); fuzzy-set qualitative comparative analysis combined with ordinary least squares regression (fsQCA+OLS); fuzzy cognitive maps combined with classification and regression trees (FCM+CRT), fuzzy failure mode and effects analysis (F-MEA).

d Dataset size. S: small; M: medium; L: large; NA: not available.

e Data sources are classified as public (Pub), institutional or hospital-based (Inst), expert-based (Exp), synthetic (Synth), or mixed.

f Comparator methods are categorized as none (no comparator), baseline (base), or direct comparison with other models (direct). Abbreviations: analytic hierarchy process (AHP); adaptive neuro-fuzzy inference system (ANFIS); artificial neural network (ANN); classification and regression tree (CRT); decision tree (DT); evolutionary programming (EP); fuzzy cognitive map (FCM); fuzzy inference system (FIS); failure mode and effects analysis (FMEA); k-nearest neighbors (kNN); learning algorithm for multivariate data analysis (LAMDA); logistic regression (LR); multilayer perceptron (MLP); naive Bayes (NB); randomized controlled trial (RCT); random forest (RF); support vector machine (SVM).

g Primary performance metrics are reported as in the original studies and include area under the curve (AUC), accuracy, sensitivity, correlation coefficients, and reliability indices. Abbreviations: Conf: confidence (association rule confidence); NR: not reported.

h Causal intent (CI) was classified as explicit (E) when formal causal inference frameworks were applied, implicit (I) when causal assumptions were suggested but not formally specified, and predictive/associative (P) when analyses focused on prediction without explicit causal interpretation.

**Table 4.** Comparative evaluations of fuzzy logic models versus conventional statistical or machine learning approaches (n=14 studies). The table summarizes studies that directly compared fuzzy-based methods with traditional models, reporting predictive performance metrics and qualitative comparative outcomes.

Study (year)	Domain	Fuzzy method	Comparators	Reported metrics	Comparative outcome
Amirkhani et al (2014) [66]	Other (AIH^a^)	NFCM^b^+NFIS^c^	NFIS, ANFIS^d^, HyFIS^e^	Acc^f^ 89.8	Neuro-fuzzy cognitive map improved explainability; performance comparable
Subramanian et al (2015) [43]	Cancer (breast)	L2-FCM^g^	Standard FCM^h^	Acc 94.3 vs 92.6	Layered FCM improved accuracy and interpretability
Yılmaz et al (2016) [45]	Cancer (lung)	ANFIS-MEP^i^	ANFIS	Acc 94.6 vs 92.6; RMSE^j^ lower	Neuro-fuzzy model achieved higher accuracy and faster convergence
Pota et al (2017) [47]	Cancer (RT^k^ side effects)	LFA^l^	Naïve Bayes	Acc 0.81 vs 0.84; mixed sensitivity/specificity	Comparable accuracy: fuzzy model offered rule-based interpretability
Stanković and Stanković (2017) [48]	Cancer (prostate)	Neuro-fuzzy	ANN^m^, FIS^n^	*R*^2^=0.83; RMSE lowest	Neuro-fuzzy slightly outperformed ANN and standard FIS
Saleh et al (2018) [41]	CVD^o^ (DR^p^)	ANFIS	RF^q^, MLP^r^, kNN^s^, DRSA^t^	Acc 84.2 vs 77.3 (DRSA)	ANFIS achieved the best accuracy among the tested classifiers
Sabahi (2018) [39]	CVD (CHD^u^ risk)	BFAHP^v^	AHP^w^	Acc 85.9 vs 77.3	Fuzzy AHP showed greater robustness under uncertainty
Argyropoulos et al (2019) [68]	AKI^x^	TSK^y^	Logistic regression	AUC^z^ 0.95 vs 0.95	Equivalent AUC; fuzzy gained sensitivity in some models
Mahmoodi et al (2020) [44]	Cancer (gastric)	FCM-NHL^aa^	ANN, SVM^ab^, DT^ac^, NB^ad^	Acc 95.8 vs 90.5 (ANN)	FCM-NHL achieved the highest predictive accuracy across methods
Yavari et al (2021) [40]	CVD (heart disease)	PB-FARM^ae^	Association rule/classification methods	Support/confidence	Fuzzy association mining extracted higher-confidence clinical rules
Mohandes et al (2022) [57]	OHS^af^ (safety)	IVIF-DEMATEL^ag^+ANP^ah^	IVIF-ANP^ai^	Reliability α=0.74	Hybrid fuzzy method prioritized causal factors with higher consistency
Benito et al (2024) [28]	ID^aj^ (COVID/dengue)	FCM+LAMDA^ak^	RF	AUC 0.89 vs 0.98 (RF)	RF outperformed in accuracy; fuzzy models offered stronger explainability
Sümbül-Şekerci et al (2024) [69]	Other (T2DM^al^ cognition)	FCM+CRT^am^	CRT	AUC 0.91 (cluster 1)	Fuzzy clustering identified cognitive subgroups; CRT supported classification
Demir and Sabır (2025) [59]	OHS	F-FMEA^an^	Classical FMEA^ao^	NR^ap^	Fuzzy FMEA reduced subjectivity in risk prioritization

a AIH: autoimmune hepatitis.

b NFCM: neuro-fuzzy cognitive map.

c NFIS: neuro-fuzzy inference system.

d ANFIS: adaptive neuro-fuzzy inference systems.

e HyFIS: hybrid fuzzy inference system.

f Acc: accuracy.

g L2-FCM: layered fuzzy cognitive map.

h FCM: fuzzy cognitive map.

i MEP: t-norm modified Einstein operator.

j RMSE: root mean square error.

k RT: radiation therapy.

l LFA: likelihood-fuzzy analysis.

m ANN: artificial neural network.

n FIS: fuzzy inference system.

o CVD: cardiovascular disease.

p DR: diabetic retinopathy.

q RF: random forest.

r MLP: multilayer perceptron.

s kNN: *k*-nearest neighbors.

t DRSA: dominance-based rough set approach.

u CHD: coronary heart disease.

v BFAHP: bimodal fuzzy analytic hierarchy process.

w AHP: analytic hierarchy process.

x AKI: acute kidney injury.

y TSK: Takagi-Sugeno-Kang.

z AUC: area under the curve.

aa NHL: nonlinear Hebbian learning.

ab SVM: support vector machine.

ac DT: decision tree.

ad NB: naïve Bayes.

ae PB-FARM: profile-based fuzzy association rule mining.

af OHS: occupational health and safety.

ag IVIF-DEMATEL: interval-valued intuitionistic fuzzy decision-making trial and evaluation laboratory.

ah ANP: analytic network process.

ai IVIF-ANP: interval-valued intuitionistic fuzzy analytic network process.

aj ID: infectious diseases.

ak LAMDA: learning algorithm for multivariate data analysis.

al T2DM: type 2 diabetes mellitus.

am CRT: classification and regression tree.

an F-FMEA: fuzzy failure mode and effects analysis.

ao FMEA: failure mode and effects analysis.

ap NR: not reported.

The most frequently addressed conditions were infectious diseases (n=10) [26-34,70], cardiovascular diseases (n=7) [35,36,38-42], cancer (n=7) [43-49], occupational health and safety (n=5) [55-59], mental health (n=2) [53,54], and preterm birth (n=2) [50,51]. Additional studies fell into miscellaneous categories [66,68,69].

Regarding data sources, most studies used institutional or hospital datasets (n=18) [31,35,36,41,44-48,50,51,55,56,66-71], while 9 relied on public datasets [28-30,32,33,38,40,49,57], 5 reported expert-based data [27,34,39,54,59], 3 reported mixed sources [26,53,58], and 2 used simulated or synthetic data [42,43].

Sample sizes varied considerably across studies: 13 used large datasets (n≥1000) [26,28-32,36,38,45,46,51,67,68], another 10 relied on medium-sized samples (n=100 to 999) [35,40,41,44,49,50,53,58,66,69], 10 used small datasets (n<100) [34,43,47,48,54-57,59,68,70], and in 4 studies, the sample size was not applicable [27,33,39,42].

A broad array of fuzzy logic techniques was identified across the included studies, reflecting substantial methodological heterogeneity. The most commonly used methods were FIS and their variations (n=8) [26,29,33,35,55,56,58,70], frequently implemented using Mamdani-type structures; followed by the FAHP (n=6) [26,31,32,39,50,67], FCM (n=5) [28,43,44,54,66,69], adaptive neuro-fuzzy systems (n=3) [41,45,48], typically combining neural architectures with fuzzy rule bases for improved learning capacity and hybrid fuzzy approach combined with multicriteria decision models (n=3) [27,38,57].

Other used models were fuzzy clustering (C or K means) (n=2) [51,67], fuzzy-trace theory (n=2) [46,49], fuzzy-set qualitative comparative analysis (fsQCA) (n=2) [30,53], Takagi-Sugeno models (n=1) [66], fuzzy failure mode and effects analysis (n=1) [59], mediative fuzzy logic (n=1) [42], fuzzy evidential reasoning (n=1) [34], likelihood-fuzzy analysis (n=1) [47], and profile-based fuzzy association rule mining (n=1) [40].

Among the studies reviewed, 14 conducted direct comparative evaluations against traditional methods such as logistic regression, decision trees, or standard statistical models [28,39-41,43-45,47,48,57,59,66,68,69] and 6 studies used baseline comparisons, typically involving simple pre/post assessments without an external benchmark [26,31,32,46,50,56]. In contrast, 17 studies applied fuzzy modeling in isolation, without any form of benchmarking or comparator method, relying solely on internal outputs to assess performance [27,29,30,33-36,38,42,49,51,53-55,58,67,70]

Between the studies that conducted direct comparative evaluations, 5 reported that fuzzy logic models outperformed traditional methods, including statistical classifiers and machine learning algorithms. These included Mahmoodi et al [44], who achieved 95.8% accuracy in gastric cancer prediction using FCM; Yılmaz et al [45], who obtained 94.6% accuracy with a neuro-fuzzy model for lung cancer; Subramanian et al [43], who reported 94.3% overall accuracy using a layered FCM for breast cancer risk; Sabahi [39], who introduced a bimodal FAHP model with accuracies above 85%; and Saleh et al [41], whose ANFIS classifier outperformed other ensemble models in diabetic retinopathy detection.

Three studies showed that fuzzy models yielded comparable or slightly superior performance relative to conventional methods. Argyropoulos et al [68] reported equivalent AUC values for both fuzzy logic and logistic regression models in predicting acute kidney injury, while Pota et al [47] found similar predictive accuracy between likelihood-fuzzy analysis and naïve Bayes classifiers in radiotherapy toxicity. Stanković and Stanković [48] also demonstrated that a neuro-fuzzy system marginally outperformed an artificial neural network in predicting prostate cancer survival.

**Figure 3.**
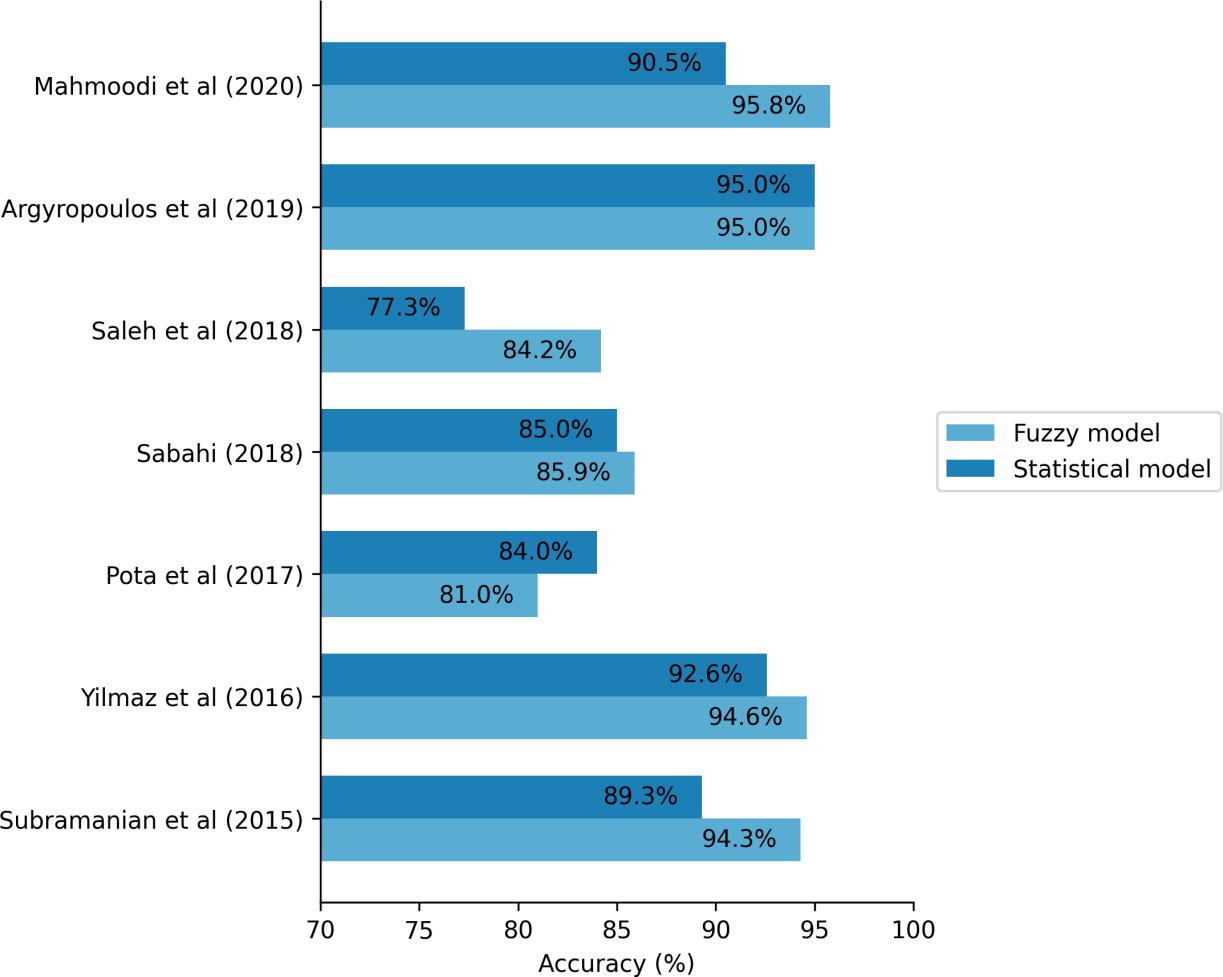
Reported accuracy values from studies performing direct quantitative comparisons between fuzzy logic–based models and conventional approaches [39,41,43-45,47,68].

The remaining 6 studies—Amirkhani et al [66], Yavari et al [40], Mohandes et al [57], Benito et al [28], Sümbül-Şekerci et al [69], and Demir and Sabır [59]—involved direct comparisons but did not report sufficient methodological or statistical detail to clearly assess the relative effectiveness of the fuzzy approach. To visually summarize the comparative performance of fuzzy logic models versus conventional statistical approaches, Figure 3 presents reported accuracy values from studies that provided quantifiable metrics. Only those studies with explicit accuracy comparisons were included, enabling a focused assessment of relative predictive performance across diverse health care domains.

Across these studies, common performance metrics included accuracy (84%‐95.8%), AUC (0.70‐0.95), and error measures such as root mean square error, mean absolute error, and mean squared error. These results underscore the adaptability of fuzzy modeling to clinical decision-making contexts marked by uncertainty, incomplete data, and the need for interpretability.

In terms of causal inference, conceptual approaches varied across the studies. While most of the studies addressed high-complexity settings involving multiple interacting variables, only two explicitly adopted formal causal inference frameworks. These included Lee et al [30], who used fsQCA with sufficiency and necessity thresholds; and Mohandes et al [57], who implemented a hybrid interval-valued intuitionistic fuzzy DEMATEL-ANP (decision-making trial and evaluation laboratory analytic network process) model with cross-validation.

Six additional studies [28,38,44,53,54,66] simulated causal mechanisms using methods such as iterative expert-based system mapping or FCM. However, none of these studies explicitly operationalized a formal causal inference framework grounded in counterfactual reasoning or directed acyclic graphs. Instead, causal assumptions were inferred through expert consensus or embedded in the structure of fuzzy systems.

The remaining 29 studies used fuzzy logic primarily for predictive or associative analysis [26,27,29,31-36,39-43,45-51,55,56,58,59,67-70], with causal relationships often left implicit, untested, or loosely derived from domain-specific knowledge alone.

Table 3 provides a detailed synthesis of the 14 studies that directly compare fuzzy logic models with traditional statistical or machine learning methods. Most of these studies reported performance gains for fuzzy approaches, particularly in cancer [43-45] and cardiovascular domains [39,41]. In several cases, fuzzy models offered not only higher accuracy or sensitivity but also enhanced interpretability. Others showed broadly comparable results with added value in robustness [47,48,68]. A smaller group reported either mixed outcomes or limited statistical detail, emphasizing interpretability and methodological novelty over raw predictive gains [28,40,57,59,66,69].

Collectively, the evidence summarized in Table 4 indicates that fuzzy logic–based approaches have been evaluated against conventional methods in a limited subset of studies, yielding heterogeneous results and variable reporting quality. Although several comparative assessments suggest potential advantages in managing uncertainty and enhancing interpretability, the absence of systematic benchmarking and the predominance of predictive objectives preclude definitive conclusions regarding comparative effectiveness.

While the findings indicate that fuzzy logic–based approaches are frequently applied in predictive health care modeling, the strength of the available evidence must be interpreted considering methodological quality and risk of bias. Seventeen studies were evaluated using the PROBAST+AI tool, specifically designed for assessing bias in prediction model studies [65]. Of these, 9 were rated as having a high risk of bias [27,34,35,39,43,44,47,66,67], and 8 studies were rated as moderate risk [28,33,40,41,45,48,68,69], standing out for more robust validation procedures, detailed variable handling, and partial transparency. None achieved a low-risk rating.

**Figure 4.**
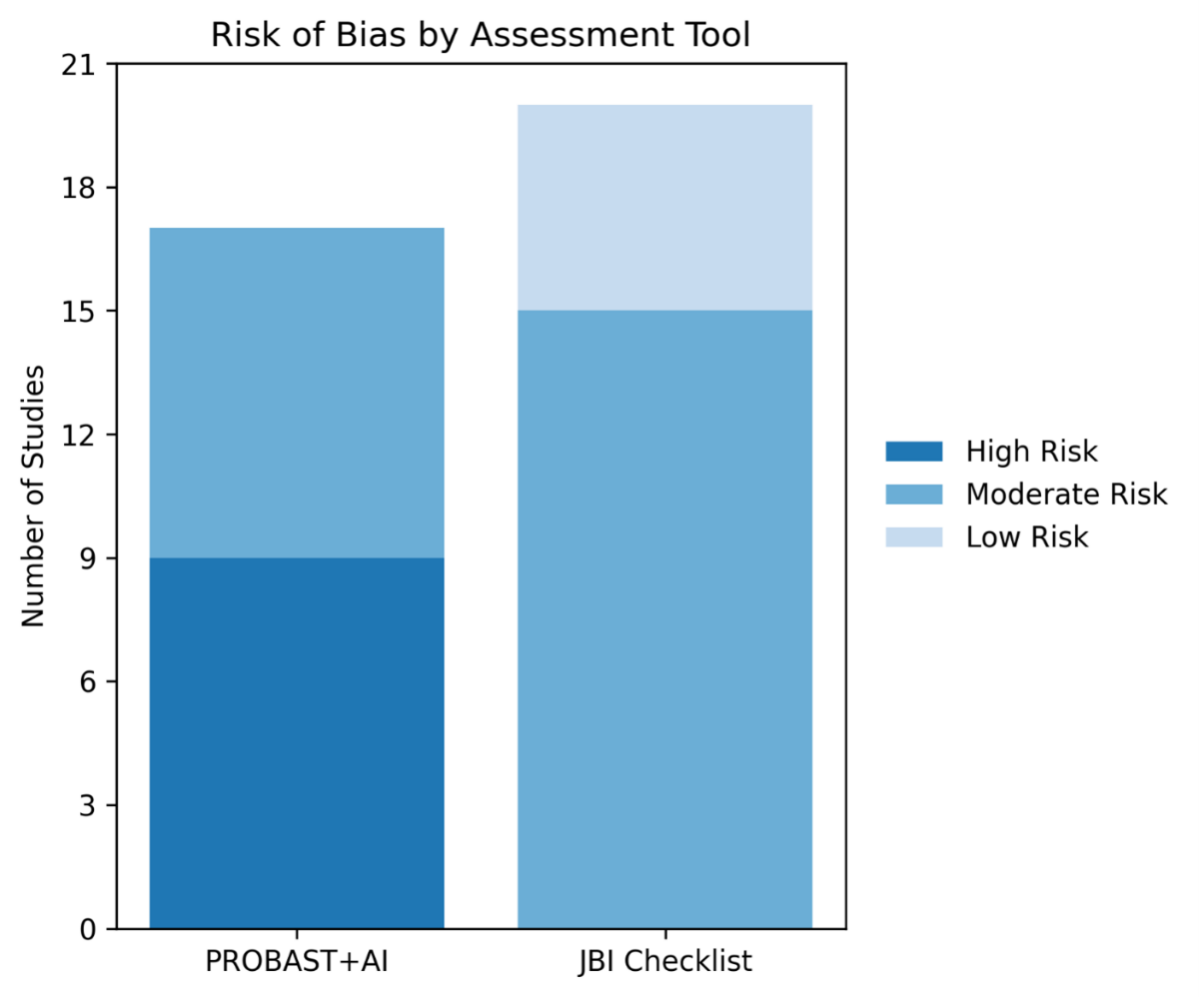
Risk of bias across included studies assessed using PROBAST+AI and the JBI checklist. Among studies evaluated with PROBAST+AI, 53% (9/17) were classified as high risk and 47% (8/17) as moderate risk, with none rated as low risk. In contrast, among studies assessed using the JBI checklist, 25% (5/20) were rated as low risk and 75% (15/20) as moderate risk. JBI: Joanna Briggs Institute; PROBAST: Prediction Model Risk of Bias Assessment Tool.

Of the 20 studies assessed using the JBI checklist [64] for analytical cross-sectional designs, 5 were rated as having low risk of bias [29,30,46,49,53], while the remaining 15 [26,31,32,36,38,42,50,51,54-59,68,70] were classified as moderate risk (Figure 4).

**Figure 5.**
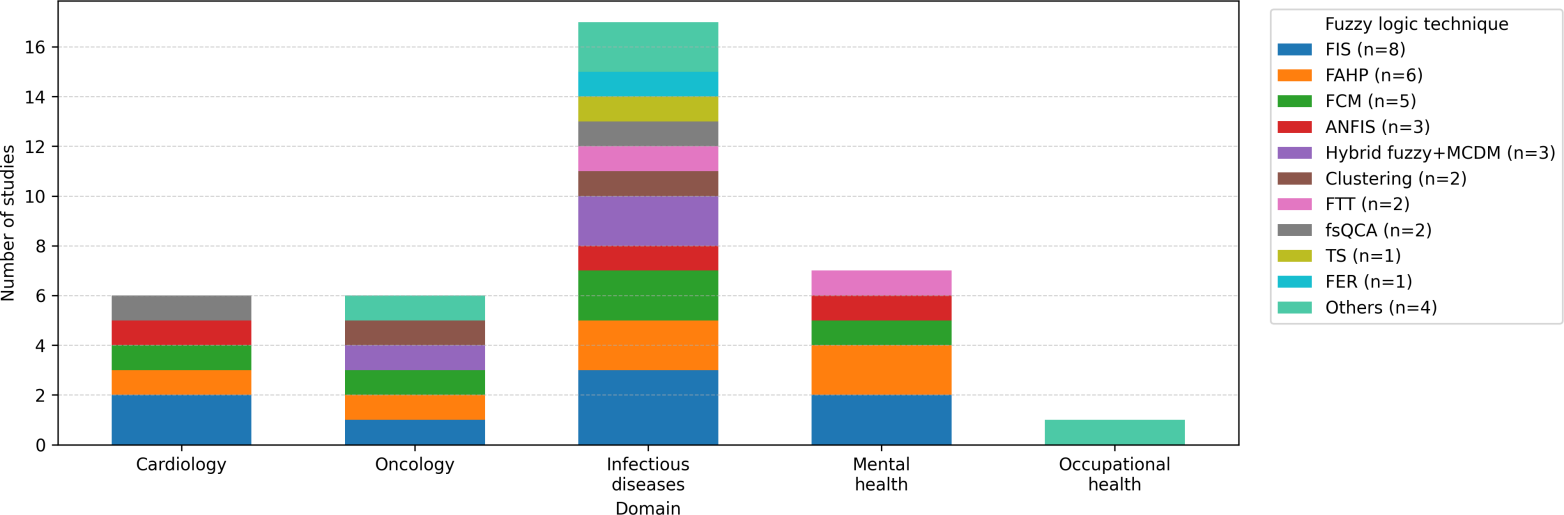
Distribution of fuzzy logic techniques across health care domains. ANFIS: adaptive neuro-fuzzy inference system; FAHP: fuzzy analytic hierarchy process; FCM: fuzzy cognitive map; FER: fuzzy evidential reasoning; FIS: fuzzy inference system; fsQCA: fuzzy-set qualitative comparative analysis; FTT: fuzzy-trace theory; MCDM: multicriteria decision-making; TS: Takagi-Sugeno model.

To examine the distribution of fuzzy logic techniques across health care applications, a cross-tabulated synthesis was conducted. As shown in Figure 5, the most frequently applied approaches were FIS, FCM, and ANFIS, followed by FAHP, fsQCA, fuzzy evidential reasoning, and Takagi-Sugeno models. The use of these techniques varied across application domains, with oncology, infectious diseases, cardiovascular health, and mental health exhibiting the highest methodological diversity.

The nature of causal engagement across the included studies spanned a continuum from explicitly formalized causal frameworks to approaches in which causal reasoning remained implicit or embedded within expert-driven or structurally defined fuzzy models. Only two studies (2/37, 5.4%) explicitly addressed causal questions using formal causal inference methodologies. A small subset relied on inferred causal structures derived from expert knowledge or fuzzy cognitive maps (6/37, 16.2%). In contrast, most studies primarily implemented predictive or associative modeling approaches, where causal interpretation was not formally specified and was instead inferred indirectly from model structure, expert judgment, or post hoc interpretation (29/37, 78.4%). This distribution highlights substantial heterogeneity in how causal principles are operationalized across fuzzy logic–based applications in health care.

## Discussion

This systematic review synthesized evidence from 37 studies published between 2014 and 2025 that used fuzzy logic–based methodologies in health care settings with explicit or implicit causal objectives. The included studies span a wide range of clinical and public health domains, including infectious diseases, cancer, cardiovascular diseases, occupational health and safety, mental health, and preterm birth, underscoring the broad applicability of fuzzy modeling to diverse health-related problems. Across domains, the most frequently reported approaches were FIS, ANFIS, FAHP, and FCM. Rather than indicating methodological convergence, this distribution reflects context-dependent adaptations of fuzzy logic to address uncertainty, nonlinearity, and expert-guided reasoning in complex health care environments.

Only a limited subset of studies conducted direct comparative evaluations between fuzzy logic–based models and conventional statistical or machine learning approaches. Among the 14 studies that included explicit comparators, 5 reported superior performance of fuzzy models [39,41,43-45]—most frequently in cancer and cardiovascular applications—while 3 demonstrated broadly comparable results [47,48,68]. The remaining 6 studies provided comparative analyses with insufficient methodological or statistical detail to support firm conclusions regarding relative effectiveness [28,40,57,59,66,67,69]. Importantly, most included studies relied on internal validation procedures, baseline comparisons, or expert-defined structures without external benchmarks, often using small- to medium-sized datasets. This pattern limits the generalizability of reported performance gains and indicates that, while fuzzy approaches may perform competitively in specific contexts characterized by nonlinearity or uncertainty, evidence supporting consistent superiority over conventional methods remains limited and heterogeneous.

Causal inference was explicitly operationalized in only a small proportion of the included studies. Specifically, two investigations adopted formal causal inference frameworks: Lee et al [30] used fsQCA, explicitly modeling configurations of necessary and sufficient conditions at the population level. Mohandes et al [57] implemented a hybrid interval-valued intuitionistic fuzzy DEMATEL-ANP approach to structurally identify and prioritize causal drivers in occupational safety systems. In both cases, causal claims were grounded in transparent methodological procedures, explicit thresholds, and internally coherent validation strategies, rather than inferred post hoc from predictive performance.

Beyond these two studies, causal reasoning was indirect. Six additional investigations relied on expert-based mappings, FCM, or influence structures to simulate causal mechanisms without formally testing necessity, sufficiency, or counterfactual dependence [28,38,44,53,54,66,69]. In most studies, fuzzy logic was applied primarily for predictive or associative purposes, with causal assumptions embedded implicitly within model architecture or domain expertise rather than explicitly articulated or empirically evaluated.

Taken together, these findings reveal a marked disconnect between the theoretical capacity of fuzzy logic to represent causal structure and its prevailing empirical use in health care research. This gap appears to reflect not inherent conceptual limitations of fuzzy methods, but rather broader issues related to study design, validation practices, and reporting rigor, which constrain the translation of fuzzy modeling from predictive decision support to explicit causal inference.

Risk of bias constituted a major limiting factor across the included studies. Among those evaluated with PROBAST+AI [65], none achieved a low-risk rating, with most classified as moderate or high risk, while only a small proportion of studies assessed using the JBI checklist [64] were rated as low risk.

In contrast to much of the existing literature, which has primarily emphasized predictive accuracy or isolated clinical applications, the present review integrates formal risk-of-bias assessment with thematic synthesis to jointly evaluate reported performance, methodological rigor, and the explicitness of causal assumptions. This perspective highlights both the strengths and current limitations of fuzzy logic–based approaches: while they provide interpretable, rule-based models well suited to ambiguity and nonlinearity, their application within explicitly causal analytical frameworks remains limited and inconsistent.

These conclusions must be interpreted considering several important limitations, including substantial heterogeneity across health care domains, modeling strategies, and outcome measures, which precluded quantitative meta-analysis; inconsistent reporting practices, such as limited use of comparator models and incomplete outcome reporting; and the frequent reliance on small- to medium-sized datasets without external validation. Collectively, these factors reduce the overall certainty and generalizability of the current evidence base.

Despite these limitations, the findings carry important implications for both research and practice. Fuzzy systems appear particularly well suited to health care and policy contexts characterized by incomplete data, multidimensional interactions, and a strong demand for interpretability. Their capacity to encode expert knowledge and tolerate imprecision supports their use in applications such as risk stratification, early diagnosis, and context-sensitive prioritization. Realizing this potential, however, will require methodological consolidation, including greater standardization in reporting, more consistent use of comparator frameworks, and external validation across real-world datasets. Importantly, integration with formal causal frameworks—such as directed acyclic graphs or structural causal models—offers a pathway to strengthen causal interpretability while preserving the distinctive advantages of fuzzy reasoning.

In parallel, recent advances in artificial intelligence have largely emphasized the automation of data extraction, measurement, and pattern recognition in clinical settings, particularly through machine learning and computer vision–based applications [71-76]. While these approaches have improved efficiency and scalability, they remain predominantly oriented toward prediction rather than causal inference. Addressing this gap requires analytical frameworks that move beyond automation to explicitly represent causal structure, intervention contrasts, and temporal assumptions.

In this context, future research would benefit from explicitly incorporating TTE [17-20] when applying fuzzy logic to observational health care data. TTE provides a principled framework for specifying causal estimands, temporal ordering, and hypothetical interventions, thereby addressing key sources of bias that remain unresolved in many fuzzy-based applications. By defining eligibility criteria, treatment strategies, follow-up periods, and causal contrasts a priori, TTE can situate fuzzy, rule-based models within transparent causal designs—an approach that is particularly relevant in real-world health care settings where randomized trials are often infeasible.

Viewed in this way, fuzzy logic should not be considered merely an auxiliary modeling technique, but a potential component of hybrid causal approaches in health care. When interpretability and causal structure are integrated into model design rather than treated as secondary considerations, fuzzy systems may help bridge the gap between statistical prediction and meaningful causal explanation. Advancing this agenda will require further methodological refinement, interdisciplinary collaboration, and a move toward more coherent and explicitly causal research programs in complex health systems.

## Supplementary material

10.2196/83425 Checklist 1 PRISMA 2020 checklist.

## ABBREVIATIONS

ANFIS: adaptive neuro-fuzzy inference system
AUC: area under the curve
DEMATEL-ANP: decision-making trial and evaluation laboratory analytic network process
FAHP: fuzzy analytic hierarchy process
FCM: fuzzy cognitive map
FIS: fuzzy inference system
fsQCA: fuzzy-set qualitative comparative analysis
GRADE: Grading of Recommendations Assessment, Development, and Evaluation
JBI: Joanna Briggs Institute
MeSH: Medical Subject Headings
PICO: population, intervention, comparator, and outcome
PRISMA: Preferred Reporting Items for Systematic Reviews and Meta-Analyses
RQ: research question
TTE: target trial emulation
