# Supplementary material for: Fuzzy Logic Approaches for Causal Inference in Health Care: Systematic Review
Source: JMIR AI. 2026 Mar 25;5:e83425. doi: 10.2196/83425 (PMC13016549; doi:10.2196/83425)
Supplement: Checklist 1 [file ai-v5-e83425-s001.pdf]

PRISMA 2020 Checklist

Section and Topic	Item #	Checklist item	Location where item is reported
**TITLE**			
Title	1	Identify the report as a systematic review.	Title page – Identified as a systematic review
**ABSTRACT**			
Abstract	2	See the PRISMA 2020 for Abstracts checklist.	Abstract – Structured abstract provided, following PRISMA for Abstracts
**INTRODUCTION**			
Rationale	3	Describe the rationale for the review in the context of existing knowledge.	Introduction – Rationale: Context of fuzzy logic in healthcare and need for causal inference
Objectives	4	Provide an explicit statement of the objective(s) or question(s) the review addresses.	Introduction – Objectives: Explicit research question and aims of the review
**METHODS**			
Eligibility criteria	5	Specify the inclusion and exclusion criteria for the review and how studies were grouped for the syntheses.	Methods – Eligibility criteria: Inclusion (2014–2025, healthcare, fuzzy logic + causal inference/predictive modeling); Exclusion (non-healthcare, non-fuzzy methods)
Information sources	6	Specify all databases, registers, websites, organisations, reference lists and other sources searched or consulted to identify studies. Specify the date when each source was last searched or consulted.	Methods – Information sources: PubMed, Web of Science, ScienceDirect (search until March 2025)
Search strategy	7	Present the full search strategies for all databases, registers and websites, including any filters and limits used.	Methods – Search strategy: Full strategies in Appendix (MeSH

Section and Topic	Item #	Checklist item	Location where item is reported
			terms, Boolean combinations)
Selection process	8	Specify the methods used to decide whether a study met the inclusion criteria of the review, including how many reviewers screened each record and each report retrieved, whether they worked independently, and if applicable, details of automation tools used in the process.	Methods – Selection process: Dual independent screening (title/abstract and full text) using ELICIT; disagreements resolved by consensus
Data collection process	9	Specify the methods used to collect data from reports, including how many reviewers collected data from each report, whether they worked independently, any processes for obtaining or confirming data from study investigators, and if applicable, details of automation tools used in the process.	Methods – Data collection process: Dual data extraction, cross-checking, third reviewer arbitration
Data items	10a	List and define all outcomes for which data were sought. Specify whether all results that were compatible with each outcome domain in each study were sought (e.g. for all measures, time points, analyses), and if not, the methods used to decide which results to collect.	Methods – Outcomes extracted: accuracy, AUC, sensitivity, specificity, RMSE, R^2
	10b	List and define all other variables for which data were sought (e.g. participant and intervention characteristics, funding sources). Describe any assumptions made about any missing or unclear information.	Methods – Other variables: sample size, domain, data source, fuzzy method, comparator, causal inference context
Study risk of bias assessment	11	Specify the methods used to assess risk of bias in the included studies, including details of the tool(s) used, how many reviewers assessed each study and whether they worked independently, and if applicable, details of automation tools used in the process.	Methods – Risk of bias: JBI checklist; PROBAST/PROBAST-AI for prediction/ML studies
Effect measures	12	Specify for each outcome the effect measure(s) (e.g. risk ratio, mean difference) used in the synthesis or presentation of results.	Methods – Effect measures: Accuracy, AUC, Sensitivity, Specificity, RMSE, R^2
Synthesis methods	13a	Describe the processes used to decide which studies were eligible for each synthesis (e.g. tabulating the study intervention characteristics and comparing against the planned groups for each synthesis (item #5)).	Methods – Eligibility for synthesis: Studies

Section and Topic	Item #	Checklist item	Location where item is reported
			grouped by domain and comparator (see Tables 2–3)
	13b	Describe any methods required to prepare the data for presentation or synthesis, such as handling of missing summary statistics, or data conversions.	Methods – Data preparation: Missing data handled narratively; no imputation performed
	13c	Describe any methods used to tabulate or visually display results of individual studies and syntheses.	Methods – Tabulation: Results tabulated in Tables 2–3 and visualized in Figures 2–3
	13d	Describe any methods used to synthesize results and provide a rationale for the choice(s). If meta-analysis was performed, describe the model(s), method(s) to identify the presence and extent of statistical heterogeneity, and software package(s) used.	Methods – Synthesis methods: Narrative synthesis; no meta-analysis
	13e	Describe any methods used to explore possible causes of heterogeneity among study results (e.g. subgroup analysis, meta-regression).	Methods – Heterogeneity: Described narratively (differences by domain, comparator)
	13f	Describe any sensitivity analyses conducted to assess robustness of the synthesized results.	Methods – Sensitivity analyses: Not applicable (no meta-analysis); qualitative cross-check only
Reporting bias assessment	14	Describe any methods used to assess risk of bias due to missing results in a synthesis (arising from reporting biases).	Methods – Reporting bias: Not applicable (no meta-analysis, no funnel plots)
Certainty assessment	15	Describe any methods used to assess certainty (or confidence) in the body of evidence for an outcome.	Methods – Certainty of evidence: Narrative discussion; GRADE not applied

PRISMA 2020 Checklist

Section and Topic	Item #	Checklist item	Location where item is reported
**RESULTS**			
Study selection	16a	Describe the results of the search and selection process, from the number of records identified in the search to the number of studies included in the review, ideally using a flow diagram.	Results – Study selection: Figure 1 (PRISMA flow diagram, 37 studies included)
	16b	Cite studies that might appear to meet the inclusion criteria, but which were excluded, and explain why they were excluded.	Results – Excluded studies: Reasons described in text/appendix
Study characteristics	17	Cite each included study and present its characteristics.	Results – Study characteristics: Table 2 (all studies) and Table 3 (comparative subset)
Risk of bias in studies	18	Present assessments of risk of bias for each included study.	Results – Risk of bias: Supplementary appendix (JBI/PROBAST tables)
Results of individual studies	19	For all outcomes, present, for each study: (a) summary statistics for each group (where appropriate) and (b) an effect estimate and its precision (e.g. confidence/credible interval), ideally using structured tables or plots.	Results – Individual study outcomes: Tables 2–3; Figures 2–3
Results of syntheses	20a	For each synthesis, briefly summarise the characteristics and risk of bias among contributing studies.	Results – Synthesis: Characteristics and risk of bias summarized narratively
	20b	Present results of all statistical syntheses conducted. If meta-analysis was done, present for each the summary estimate and its precision (e.g. confidence/credible interval) and measures of statistical heterogeneity. If comparing groups, describe the direction of the effect.	Results – Statistical syntheses: Not applicable (no meta-analysis); comparative summary in Table 3
	20c	Present results of all investigations of possible causes of heterogeneity among study results.	Results – Heterogeneity results: Reported narratively (variation across domains, comparators)

Section and Topic	Item #	Checklist item	Location where item is reported
	20d	Present results of all sensitivity analyses conducted to assess the robustness of the synthesized results.	Results – Sensitivity analyses: Not applicable (no meta-analysis); narrative robustness check
Reporting biases	21	Present assessments of risk of bias due to missing results (arising from reporting biases) for each synthesis assessed.	Results – Reporting biases: Not assessed (no meta-analysis)
Certainty of evidence	22	Present assessments of certainty (or confidence) in the body of evidence for each outcome assessed.	Results – Certainty of evidence: Narrative synthesis; heterogeneous evidence base
**DISCUSSION**			
Discussion	23a	Provide a general interpretation of the results in the context of other evidence.	Discussion – Interpretation: Results contextualized with prior literature
	23b	Discuss any limitations of the evidence included in the review.	Discussion – Limitations of evidence: Quality and heterogeneity of included studies
	23c	Discuss any limitations of the review processes used.	Discussion – Limitations of review: No meta-analysis; causal integration inconsistently reported
	23d	Discuss implications of the results for practice, policy, and future research.	Discussion – Implications: Implications for practice, policy, and future research
**OTHER INFORMATION**			
Registration and protocol	24a	Provide registration information for the review, including register name and registration number, or state that the review was not registered.	Other info –

PRISMA 2020 Checklist

Section and Topic	Item #	Checklist item	Location where item is reported
			Registration: Not registered
	24b	Indicate where the review protocol can be accessed, or state that a protocol was not prepared.	Other info – Protocol access: No protocol prepared
	24c	Describe and explain any amendments to information provided at registration or in the protocol.	Other info – Amendments: Not applicable (no registration)
Support	25	Describe sources of financial or non-financial support for the review, and the role of the funders or sponsors in the review.	Other info – Support: ANID Doctoral Scholarship; institutional support (University of Valparaíso)
Competing interests	26	Declare any competing interests of review authors.	Other info – Competing interests: None declared
Availability of data, code and other materials	27	Report which of the following are publicly available and where they can be found: template data collection forms; data extracted from included studies; data used for all analyses; analytic code; any other materials used in the review.	Other info – Availability: Extraction tables, coding, and figures available upon request; supplementary materials

From: Page MJ, McKenzie JE, Bossuyt PM, Boutron I, Hoffmann TC, Mulrow CD, et al. The PRISMA 2020 statement: an updated guideline for reporting systematic reviews. BMJ 2021;372:n71. doi: 10.1136/bmj.n71. This work is licensed under CC BY 4.0. To view a copy of this license, visit https://creativecommons.org/licenses/by/4.0/
